# Elekta Unity MR-linac commissioning: mechanical and dosimetry tests

**DOI:** 10.1093/jrr/rrac072

**Published:** 2022-11-14

**Authors:** Masato Tsuneda, Kota Abe, Yukio Fujita, Yohei Ikeda, Yoshinobu Furuyama, Takashi Uno

**Affiliations:** Department of Radiation Oncology, MR Linac ART Division, Graduate School of Medicine, Chiba University, Chiba, 260-8677 Japan; Department of Radiation Oncology, MR Linac ART Division, Graduate School of Medicine, Chiba University, Chiba, 260-8677 Japan; Department of Radiation Oncology, MR Linac ART Division, Graduate School of Medicine, Chiba University, Chiba, 260-8677 Japan; Department of Radiation Sciences, Komazawa University, Setagaya, Tokyo, 259-1193 Japan; Department of Radiology, Chiba University Hospital, Chiba, 260-8670 Japan; Department of Radiology, Chiba University Hospital, Chiba, 260-8670 Japan; Diagnostic Radiology and Radiation Oncology, Graduate School of Medicine, Chiba University, Chiba, 260-8677 Japan

**Keywords:** commissioning, mechanical QA, dosimetry, MR-linac, Elekta Unity, off-center Winston-Lutz (WL) test

## Abstract

We report the commissioning results of Elekta Unity for the dosimetric performance and mechanical quality assurance (QA), and propose additional commissioning procedures. Mechanical tests included multi-leaf collimator (MLC) positional accuracy, radiation isocenter diameter at the center and off-center position, and coincidence between the magnetic resonance (MR) image center and radiation isocenter. Comparisons between the measurements and calculations of the simple irradiated field, intensity modulated radiation therapy (IMRT) commissioning, MLC output factor ratio, validation of independent dose calculation software and end-to-end testing were performed to evaluate dosimetric performance. The average values of the MLC positional accuracy for film- and imaging device-based analysis were −0.1 and 0.3 mm, respectively. The measured radiation isocenter size was 0.41 mm, and the off-center results were within 1 mm. The coincidence was −0.21, −1.19 and 0.49 mm along the x-, y- and z-axes, respectively. The calculated percent depth doses (PDD) and profiles agreed with the measurements. The results of independent dose calculation were within the action level recommended by American Associations of Physicist in Medicine. The gamma passing rate (GPR) for IMRT commissioning was 98.6 ± 0.9%, and end-to-end testing of adapted plans showed agreement within 2% between the measurement and calculation. We reported the results of mechanical and dosimetric performances of Elekta Unity, and proposed novel commissioning procedures. Our results should provide knowledge to the physics community for enhancing the QA programs.

## INTRODUCTION

Magnetic resonance (MR) images have often been used for precise delineation of the target and organs-at-risk. Recently, MR guided online adaptive radiation therapy (MRgOART) gained momentum due to two advantages: the ability to repeat 3-dimensional imaging and intrafraction cine imaging without additional radiation exposure [1–4], and another ability to adapt an offline intensity modulated radiation therapy (IMRT) plan to the patient anatomy at each treatment fraction.

An increasing number of quality assurance (QA) and commissioning articles for MRgOART systems have been reported worldwide [5–8]; however, no comprehensive QA guidelines have yet been published by major societies such as American Association of Physicists in Medicine (AAPM), International Atomic Energy Agency (IAEA), European Society for Radiotherapy and Oncology to name a few. The purpose of this article was to provide our commissioning results and additional QA proposals to the community thereby enriching the QA programs and possibly accelerating the publication of the guideline. In more details, we have proposed, for the first time, an off-center Winston-Lutz test (WL test) as an additional machine QA procedure to verify delivery accuracy of non-isocentric treatment fields that are often employed by our 1.5 T MR-linac. We have also performed independent dose QA using a commercial software, MU2net and an inhomogeneous phantom in accordance with AAPM Task Group 219 [9]. To the author’s knowledge, the MU2net-based QA results has been reported only with a homogeneous phantom, and therefore the present article may provide additional knowledge to the physics community.

**Fig. 1:**
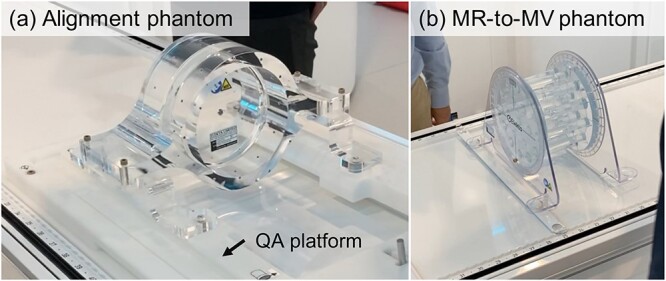
Setup of (a) alignment and (b) MR-to-MV phantoms.

## MATERIALS AND METHODS

### Overview of Elekta Unity

The Elekta Unity system comprises a 1.5 T Philips MR imaging system (Philips Healthcare, Amsterdam, the Netherlands) and a 7-megavoltage (MV) flattening filter-free linear accelerator [4]. The beam generation system is mounted on a slip-ring gantry that smoothly rotates around a cylindrical cryostat containing the 1.5 T superconducting magnet. The cryostat is a thermally insulating enclosure storing liquid helium. Elekta Unity’s cryostat has a thin annulus or a pipe containing the liquid helium at the beam passing region. The annulus connects the cryostat in the longitudinal direction, thereby minimizing beam attenuation caused by the liquid helium. The cryostat cross-over pipe provides electrical connection between the split coils. Unity has a 160-leaf multi-leaf collimator (MLC) moving in the longitudinal direction. The MLC shapes radiation fields ranging from 0.5 × 0.5 cm^2^ to 57.4 × 22.0 cm^2^ on the isocenter plane. In addition, Unity has an MV imager (MVI) with an imaging dimension of 22 × 9.5 cm^2^, which is used for the MLC leaf calibration and ionization chamber alignment. The source-to-isocenter distance of the Unity beamline is 143.5 cm, which is longer than that in the conventional linacs. The patient couch moves only in the longitudinal direction; and therefore, treatment fields are defined away from the isocenter according to the target position in the patient body. Consequently, the accuracy for non-isocentric beam delivery is important, and needs to be verified.

### Mechanical

#### Radiation isocenter diameter

To measure the radiation isocenter diameter, Elekta developed an alignment phantom. This phantom was placed on the QA platform, and the phantom center was aligned as the isocenter position. These tools were specialized for Elekta Unity, which are shown in Fig. 1 (a). The alignment phantom had a large ball-bearing (BB) at its center. The irradiated field was 5 × 5 cm^2^ with a delivered dose of 100 monitor unit (MU). This field was formed using only the MLC, i.e. without X-diaphragm. The photon beams were irradiated at 30° intervals from 0 to 360°, excepting 90° and 270°. The WL algorithm was employed to determine the radiation isocenter diameter.

**Fig. 2:**
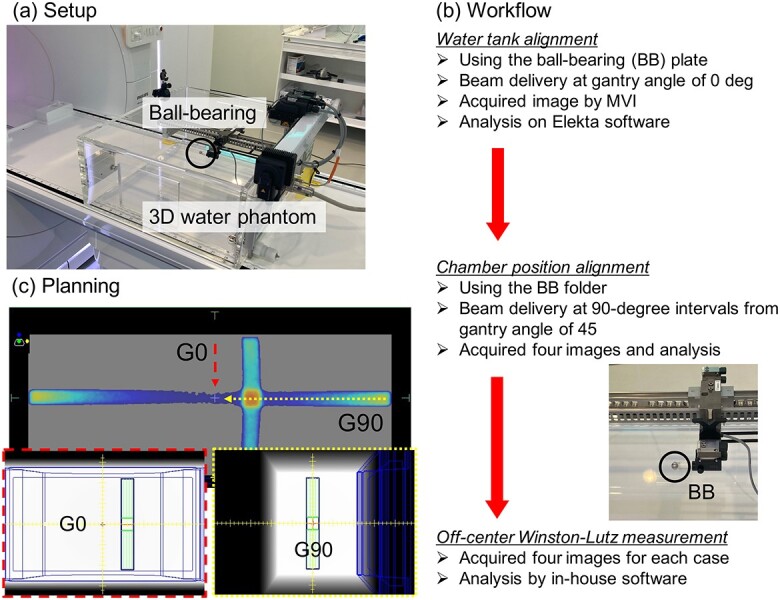
(a) Photograph of measurement setup for off-center WL test, (b) measurement workflow and (c) picture of planning screen in the case of an offset along the lateral direction by 6 cm.

In addition, we developed an off-center WL test (off-WL test) for Unity commissioning. A 3D scanning water phantom (PTW, Freiburg, Germany) was used. Figure 2 (a) and (b) show the measurement setup and workflow, respectively. First, the water phantom was aligned by placing a BB plate placed at the bottom of the tank. MV image that irradiated from gantry angle of 0 degrees was acquired and analyzed using the water phantom alignment software provided by Elekta. Subsequently, we aligned the chamber position using 3D coordinate. The BB was attached to the chamber folder, and MV images were acquired at gantry angles of 45, 135, 225 and 315°. These images were analyzed to detect the BB position. After these alignments, the BB was at the isocenter and the scan reference center in water phantom coordinate. We created an irradiation plan with non-isocentric beams (Fig. 2 [c]). Fields of these non-isocentric plans were created using the MLC and X-diaphragm to form a 3 × 3 cm^2^. The MV images were acquired for each beam. Moreover, we also verified the positional accuracy of the BeamSCAN-MR system. MV images were acquired for each case of the BB offset, and the BB positions were detected. These steps were repeated five-times to evaluate the differences between the measured and actual values.

#### Coincidence between the MR image center and radiation isocenter

The distance between the MR image center and radiation isocenter was measured to evaluate these centers coincidence. The MR-to-MV measurement phantom developed by Elekta is shown in Fig. 1 (b). This phantom comprised a bottle that was formed using a known geometry, seven Zirconia spheres and a copper sulfate solution. The spheres were projected on MVI, and signals of spheres were voided in MR images. The centers of all spheres were detected in both imaging modalities, and the detected positions were compared to calculate the coincidence. The irradiated plan, MR acquisition sequence and analysis software were provided by Elekta.

#### MLC alignment

Two fence tests were performed using the MVI and gafchromic films (EBT-3, Ashland, NJ, USA). Unfortunately, the detectable region of the MVI was 22 × 9.5 cm^2^; therefore, only the central 28 leaf pairs could be captured. In this study, a film-based fence test was also performed to capture the positions of all leaf pairs. However, because the fully open irradiated fields at a gantry angle of 0° interrupt the pipe, the test cannot be performed for all 80 MLC pairs. In the present study, 70 pairs of MLCs were described as all MLCs. Three, open strips with a width of 10 mm were created at positions −30, 0 and +30 mm. A film inserted between copper plates with a thickness of 3 mm was placed on the isocenter plane. To detect all MLCs, the two films were combined and marked. The films were digitized using an Epson 12000XL scanner at 150 dpi (0.169 mm/pixel), and the processing was automated using in-house software to identify the location of the exposed strips from the resulting profiles.

### Dosimetry

#### Calibration and Beam quality

Reference dose calibration was performed using a farmer-type chamber (Exradin A19-MR, Standard Imaging, WI, USA) located in a water phantom (MP1; PTW, Freiburg, Germany). O’Brien *et al*. proposed the use of a chamber-specific *k_B_* factor that accounts for changes in the chamber response owing to the magnetic field [10]. The measurements were performed with the chamber aligned parallel to the magnetic field and at a gantry angle of 0°. A gantry angle of 90° was used to exclude the changes in helium levels in previous studies. However, a gantry angle of 0° is used in our institute because there are no facilities with helium levels above 93.4%, and the water-equivalent thickness of the polymethyl methacrylate (PMMA) must be considered when the gantry angle is 90°. Currently, Elekta recommends a gantry angle of 0° for dose calibration. The position of the chamber at the isocenter was adjusted using the orthogonal MV images. It has been reported that the *%dd(10)x* factor changes depending on the presence or absence of a magnetic field. Therefore, tissue phantom ratio_20, 10_ (TPR_20,10_) was measured to measure the beam quality [10]. Unity was designed to have a fixed-dose rate of 425 MU/min at the calibration depth. In this study, a calibration depth of 5 cm was selected instead of *d_max_*.

**Fig. 3:**
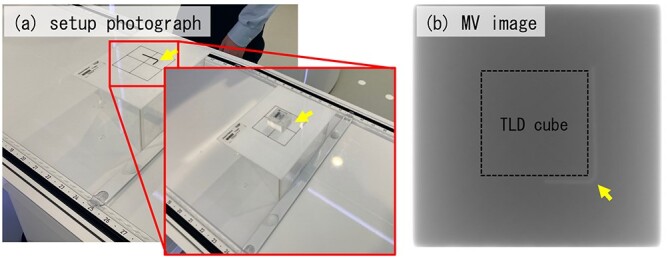
Setup photograph (a) and measured image for measurement setup (b). Yellow arrows indicate the corner of TLD cube phantom.

Independent validation provided by the MD Anderson Cancer Center Radiation Dosimetry Services (MDACC RDS) was examined. The thermoluminescent dosimeters (TLD) were irradiated with 300 MU and then mailed to the MDACC RDS for reading. For the measurement setup, wires were placed on the corner of the TLD cube phantom, and MV images was acquired by MVI (Fig. 3). After the setup, the wires ware replaced to the TLD.

#### Beam data collection

All measurements were performed using the BeamSCAN-MR. The percent depth doses (PDDs) and profiles were acquired according to the beam data collection list provided by Elekta. Owing to the limitations of the Unity bore and the water phantom dimensions, the maximum scan depth was 15 cm when the gantry angle of 0°. At the gantry at 270°, the maximum scan depth was 38 cm; however, the maximum field size was 16 × 16 cm^2^, depending on the size of the thin PMMA window. PDDs and profiles for field sizes of 2 × 2, 3 × 3, 5 × 5, 10 × 10, 15 × 15, 22 × 22, 40 × 22, 53.5 × 22 and 57.4 × 22 cm^2^ were acquired with the gantry set to 0°, and those for field sizes of 2 × 2, 3 × 3, 5 × 5, 10 × 10 and 16 × 16 cm^2^ were acquired with the gantry set to 270°. A PTW 0.07 cc ionization chamber (TN31021) and PTW microdiamond detector (TN60019) were used. Measurements were compared to the TPS calculation using a gamma analysis of 2% dose difference (DD) and 2 mm distance to agreement (DTA) with global normalization. For the comparison, the Monaco commissioning utility software was used for the gamma analysis. As part of the commissioning process, it is necessary to measure the attenuation correction of the cryostat, which requires the removal of the posterior coils and bridge from the couch so that measurements are not affected. This is because the construction of the cryostat was not uniform over the surface of the magnet. An ionization chamber in a build-up cap was placed such that the chamber center was at the radiation isocenter. This chamber was aligned using the acquired MV images (0, 90, 180 and 270°). After alignment, the output for a field size of 10 × 10 cm^2^ were measured every 2°. The reading values of the chamber were normalized to a 90° gantry angle to model the cryostat attenuation correction in the Monaco for Unity treatment planning system (TPS).

#### Gantry angle dependency

The relationship between the output and gantry angle was influenced by the presence of the MR-linac couch and the accuracy of the cryostat correction. To evaluate the gantry angle dependency, the ArcCHECK (Sun Nuclear, FL, USA) was set up on the QA platform. The ionization chamber was positioned at the center of the ArcCHECK. Charge readings were obtained for a 10 × 10 cm^2^ field size at different gantry angles and normalized to the 90° gantry angle.

#### Open and MLC fields dose measurement

We prepared a conventional plan to evaluate calculation accuracy. The calculated doses were acquired from these plans. We also created MLC fields, e.g. circular, oval, irregular, V-shaped and asymmetric field plans. The 10 × 10 cm^2^ field was moved ±5 cm in the x- and y-directions to create a total of eight asymmetric field plans. Absolute dose for the field sizes of 2 × 2, 3 × 3, 4 × 4, 5 × 5, 8 × 8, 10 × 10, 15 × 15, 20 × 20, 22 × 22, 6 × 10, 10 × 6, 8 × 15 and 15 × 8 cm^2^ were measured at depths of 1.3, 5, 10 and 15 cm. A19 and A1SL (Standard Imaging, WI, USA) ionization chambers were used for large (≥ 5 × 5 cm^2^) and small fields (< 5 × 5 cm^2^), respectively. The measured dose was compared to the calculated dose.

#### Independent dose calculation

Modeled MU2net (DOSIsoft, France) software was implemented for independent dose calculation. The independent dose calculation using this software does not consider the magnetic field, i.e. *B* = 0 T environment. Absolute doses per 100 MU at a depth of 10 cm were registered, and the calculated dose determined the absorbed dose in water. To evaluate the calculation accuracy, the calculated values for MU2net were compared with the measurements. Field size was ranged from 2.0 × 2.0 cm^2^ to 22.0 × 22.0 cm^2^. The calculation accuracy of the square fields at the off-isocenter position was also evaluated. An asymmetric field plan was also used, as described in 2.2.4. The A1SL detector was used for field sizes less than 5 × 5 cm^2^, and the A19 was used for field sizes of at least 5.0 × 5.0 cm^2^.

The accuracy of the independent dose calculation in the heterogeneous phantom was verified using a water tank-type heterogeneous lung phantom [11]. The irradiation fields were shaped with MLC margins of 0.3, 0.5 and 1.0 cm for the tumor. The gantry angle was 0 degrees, and the four-field conventional and the seven-field IMRT plans were also verified. For the IMRT plan, the prescribed dose for the target was 42 Gy in four fractions.

#### MLC output factor ratio

Subashi *et al*. proposed that dynamic MLC measurements to evaluate the dosimetric accuracy of IMRT deliveries [8]. To quantify the accuracy and stability of the MLC model in the TPS, the A19 chamber was inserted into the solid water phantom at the isocenter at a depth of 5 cm. The width of MLC was 1 cm, and 20 MU was delivered for each segment. The MLC travel length was 10 cm along the longitudinal axis; therefore, the segmental MLC IMRT (S-IMRT) plan consisted of 11 segments. An open field (10 × 10 cm^2^) was irradiated with dose of 220 MU. The measurement was normalized to the output of the open field to generate the MLC output factor ratio. The reproducibility of the measurements was also verified. There was correlation between the MLC gap width and measured MLC output ratio. We created an irradiation plan with a gap error ranging from 0.1, 0.2, 0.3, 0.4, 1.0 and 2.0 mm. The MLC output ratio with gap error was also measured and compared with the correlation.

#### IMRT commissioning

The symmetry and reproducibility of the irradiated fields were verified for the delivery accuracy of the S-IMRT fields. A 2-dimensional array detector, IC profiler-MR (Sun Nuclear, FL, USA), was used. The profiles and the central axis (CAX) dose delivered with a low dose (3 MU) were compared with those delivered with a dose of 200 MU.

IMRT treatments were commissioned using data sets from the AAPM Task Group 119, AAPM Medical Physics Practice Guideline, and prostate plan at our institute [12, 13]. In total, 11 data sets were measured, and the measurements were compared with calculations using gamma analysis. Gamma analysis of 3% DD and 2 mm DTA with global normalization was performed for all cases in accordance with the AAPM TG-218 guideline [14]. The ArcCHECK and QA platform were contoured to apply relative electron density (rED) overrides. The QA platform was assigned a rED of 1.2. This value was recommended by Elekta. And, the rED of the ArcCheck for use with the TPS was 1.16. These treatment plans were used to calculate the dose distributions delivered to the ArcCHECK with 3 mm dose grid and a 1% uncertainty per calculation. Point-dose measurements were performed using ArcCHECK for each data set.

#### End-to-end testing

End-to-end tests were performed using a CIRS phantom (model 008Z; VA, USA). The phantom includes the liver, kidney, vertebra, spinal cord, lung and ionization chamber insert. The tumors were located in the kidney and liver, and the chamber could be inserted in these positions. Computed tomography (CT) and MR images were obtained for treatment planning. A 2-min T2-weighted 3D image was selected from the exam card list. The images were then transferred to the TPS. Some organ structures were delineated on the CT images, and the mean rED values were acquired. CT images were registered to the MR images, and a step-and-shoot IMRT plan was created on MR images with the structures and rED information. This plan prescribed a dose of 40 Gy in five fractions. Dose calculation was performed with a 3-mm dose grid, with 1% statistical uncertainty per calculation.

The phantom was positioned on the treatment couch, and daily-MR images were acquired. The images were registered to the initial MR images. Adapt-to-position (ATP) plan and adapt-to-shape (ATS) plans were created. When performing plan adaptation methods using optimize weights and shapes, the original segments were discarded, and new initial plan segmentation was performed [15]. Before the irradiation, the independent dose calculation was performed. The ionization chamber was inserted into the liver and kidney position of the phantom. The A1SL chamber was calibrated against a farmer-type reference-ion chamber. A small amount of water was added to the insert owing to the air gaps between the chamber and phantom. The measured doses were recorded for each adaptation plan. The measurement was compared against the calculated point dose (mean dose in the volume of interest). These adaptation plans were used to calculate the dose distributions delivered to the ArcCHECK.

#### Result of patient-specific QA

From December 2021 to January 2022, 2 kidney, 5 liver, 2 pelvic and 1 pancreatic cancer patients were treated with MRgOART at our institution. The initial and adaptation plans were used to calculate the dose distributions delivered to the ArcCHECK. The measurements were compared with the calculations using the gamma analysis. In cases where it was measurable, the point dose was measured and evaluated.

## RESULTS

### Mechanical

#### Radiation isocenter

The result for the radiation isocenter was 0.41 mm. The recommended tolerance was within 0.5 mm [5]. Our result was within the tolerance range. Powers *et al*. reported that the radiation isocenter was measured using a combination of multiple phantoms and analysis software [7]. All the results were within 0.5 mm. The slip-ring design of Unity allowed for a very tight isocenter in comparison with the standard linac.

The average difference and SD were (0.03 and 0.01), (−0.09 and 0.02) and (−0.04 and 0.02) mm in the x, y and z axes, respectively. The positional accuracy of BeamSCAN-MR system was thus confirmed. Table 1 shows the results of the radiation isocenter using the alignment phantom and BeamSCAN-MR. Robert *et al*. reported that the use of a radiation field with fixed collimator components, such as leaf sides, is advisable in the case of systems without collimator rotation [5]. The discrepancy owing to the positioning accuracy of the X diaphragm can be removed. The 5 × 5 cm^2^ irradiated field provided by Elekta was the irradiated field formed only by the MLC. Irradiated fields (3 × 3 cm^2^) for verifying the off-center radiation isocenter were formed by MLCs and an X-diaphragm. Hence, the result of the off-center radiation isocenter includes an uncertainty in the accuracy of the X-diaphragm.

**Table 1:** Results of radiation isocenter

Setup		Tools	Field size	Radiation isocenter [mm]
Isocenter		Alignment phantom	5 × 5	0.41
Isocenter		BeamSCAN-MR	3 × 3	0.54
Off-center [mm]	x + 30	BeamSCAN-MR	3 × 3	0.58
	x + 60	BeamSCAN-MR	3 × 3	0.63
	y + 15	BeamSCAN-MR	3 × 3	0.54
	z + 30	BeamSCAN-MR	3 × 3	0.59

#### Coincidence between the MR image center and radiation isocenter

The coincidence was −0.21, −1.19 and 0.49 mm in the x-, y- and z-axes, respectively. This translation parameter was applied to the TPS for the plan adaptation. Tolerance of this coincidence was not recommended; however, the trend must be verified.

#### MLC alignment

**Fig. 4:**
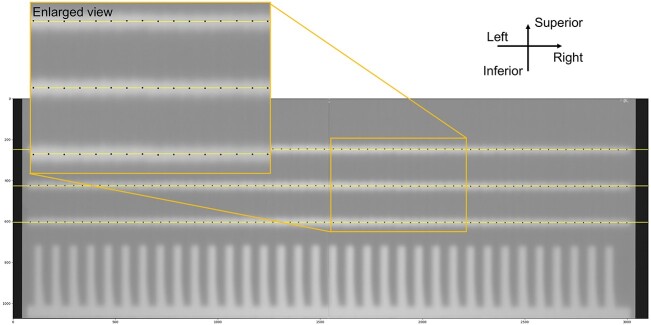
Result of the film-based fence test.

For MVI-based measurement, the average and standard deviation (SD) for the MLC position were 0.3 ± 0.3 mm. For film-based measurement, the average and SD were −0.1 ± 0.3 mm. Figure 4 shows the analyzed film with the detected positions and centerlines.

### Dosimetry

#### Calibration and beam quality

The TPR_20,10_ value was 0.705. Snyder *et al*. and Powers *et al*. reported TPR_20,10_ values of 0.704 and 0.703, respectively [6, 7]. The k_q_ factor from IAEA technical reports series 398 was 0.989. Markov *et al*. reported k_B_ factor for the A19 chamber oriented parallel with respect to the magnetic field was 1.0007 [16]. We used this value as the k_B_ factor. Calibration was performed with a source-to-axis distance setup which consisted of a source-to-surface distance of 138.5 cm and a depth of 5 cm. The dose calibrated by the institution in a water phantom at a depth of 5 cm was 300 cGy. In contrast, the dose measured by the MDACC was 305 cGy; therefore, the ratio of the dose by the MDACC to that of our institute was 1.02. An agreement within ±5% was considered a satisfactory check.

#### Beam data collection

**Fig. 5:**
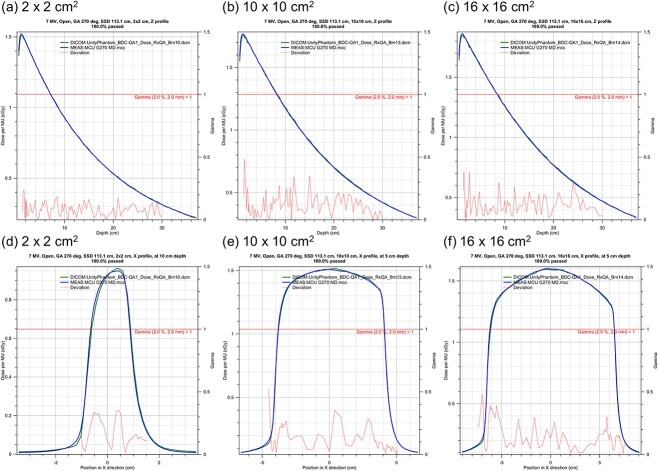
PDDs and profiles measured at a gantry angle of 270°. (a) 2 × 2 cm^2^, (b) 10 × 10 cm^2^, and (c) 16 × 16 cm^2^ PDDs and (d) 2 × 2 cm^2^, (e) 10 × 10 cm^2^, and (f) 16 × 16 cm^2^ cross line profiles.

**Fig. 6:**
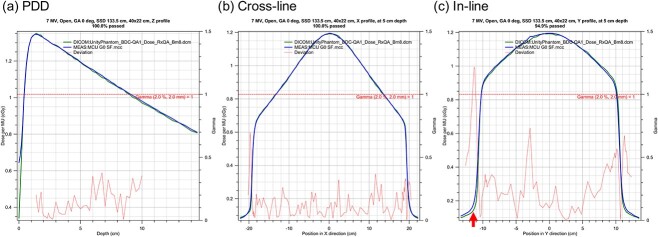
PDDs and profiles for a large field size (40 × 22 cm^2^) measured at a gantry angle of 0°.

**Fig. 7:**
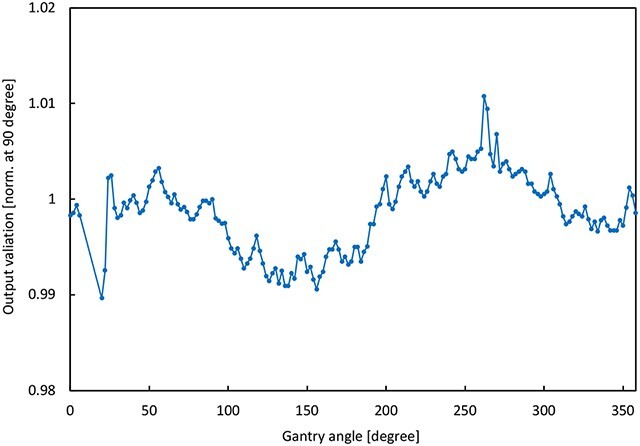
Cryostat attenuation of gantry dependency.

Figure 5 shows representative PDDs and profiles measured at a gantry angle of 270°. The measured PDDs and profiles were compared with the calculation using the gamma analysis (2%/2 mm). All the gamma passing rates (GPRs) were 100%. Figure 6 shows PDDs and profiles for a large field size measured at a gantry angle of 0°. The average and minimum GPRs were 99.6% and 94.6%, respectively. The case of the minimum GPR was a profile with the irradiated field of 40 × 22 cm^2^ and a depth of 1.3 cm. In general, gamma values above 1 were only seen in the tail region for the largest measured field sizes. The red arrow in Fig. 6(c) indicates the gamma value above 1. The cryostat attenuation is shown in Fig. 7. The results were within 1%, except those for the gantry angle 20° and 262°.

#### Gantry angle dependency

**Fig. 8:**
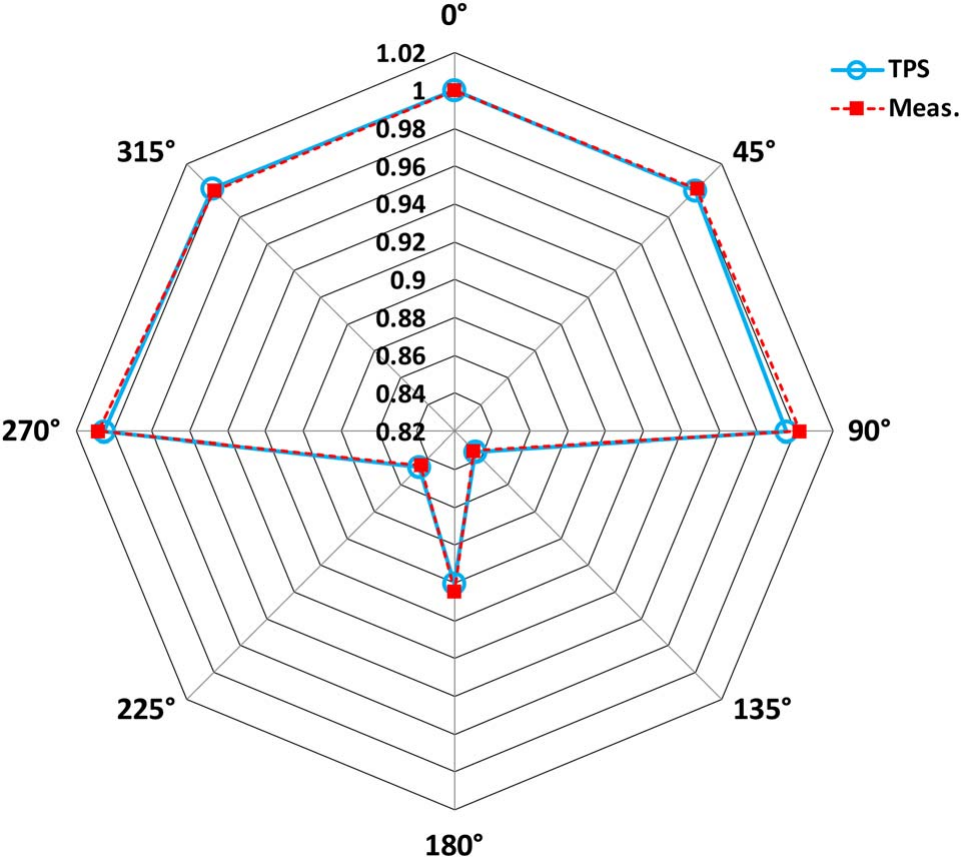
Gantry angle dependency.

Figure 8 shows the gantry angle output dependency between the measurement and TPS calculation. The maximum and average of the difference was −0.7% and −0.2%, respectively. The measurement was in good agreement with the calculation considering cryostat correction and couch attenuation.

#### Open and MLC fields dose measurement

**Fig. 9:**
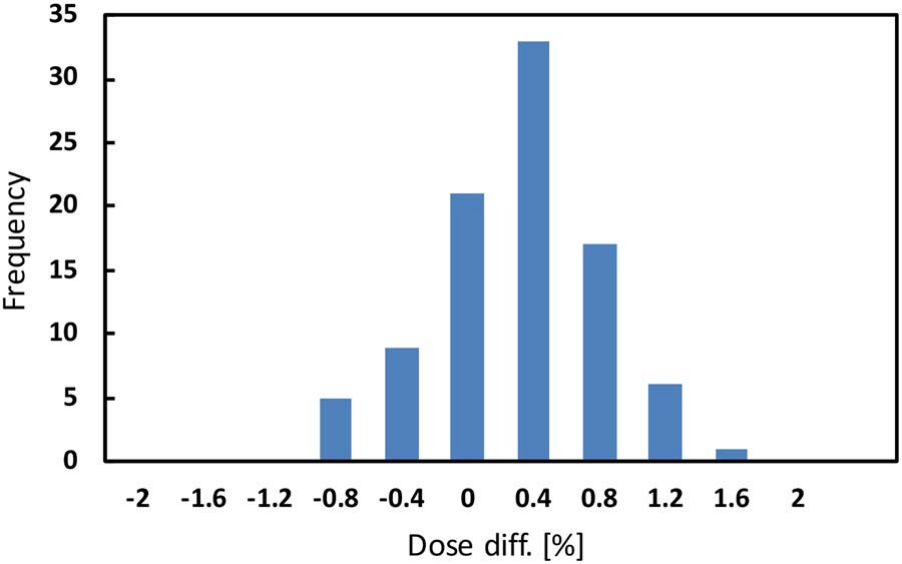
Histogram of DD between the calculation and the measurement.

Figure 9 shows a histogram of the DD between the measurement and calculation. The average and maximum of DDs were 0.11% and 1.21%, respectively, confirming the accuracy of the calculations.

#### Independent dose calculation

**Fig. 10:**
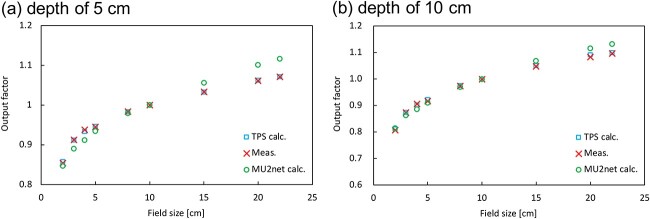
Output factor for TPS and MU2net calculations and measurements at depths of (a) 5 cm and (b) 10 cm.

The measured doses for the square fields were normalized to the dose for 10 × 10 cm^2^ fields. The calculation by MU2net was compared with the measurement and calculation by the TPS. Figure 10 shows the output factors for the measurements and calculations. The measurement and TPS calculation for both depths were within 0.7% for all the field sizes. In the comparison of measurements and MU2net calculations, the maximum DD for depths of 5 cm and 10 cm was 4.2% and 3.2%, respectively. The maximum differences were observed for the 22 × 22 cm^2^ field. The average dose error of the non-isocentric irradiated fields was 2.32%; however, the dose errors for the 10 × 10 cm^2^ field at the CAX were 1.5% and 0.5% for the 5 cm and 10 cm depths, respectively. The dose error in the non-isocentric field was insignificant.

Table 2 and 3 shows the results of calculation accuracy of single beams, conventional plan and IMRT plan. All differences were within 5% and 7% for composite plan and single beam, respectively. This criterion was recommended by AAPM Task Group 219 [9].

#### MLC output factor ratio

**Fig. 11:**
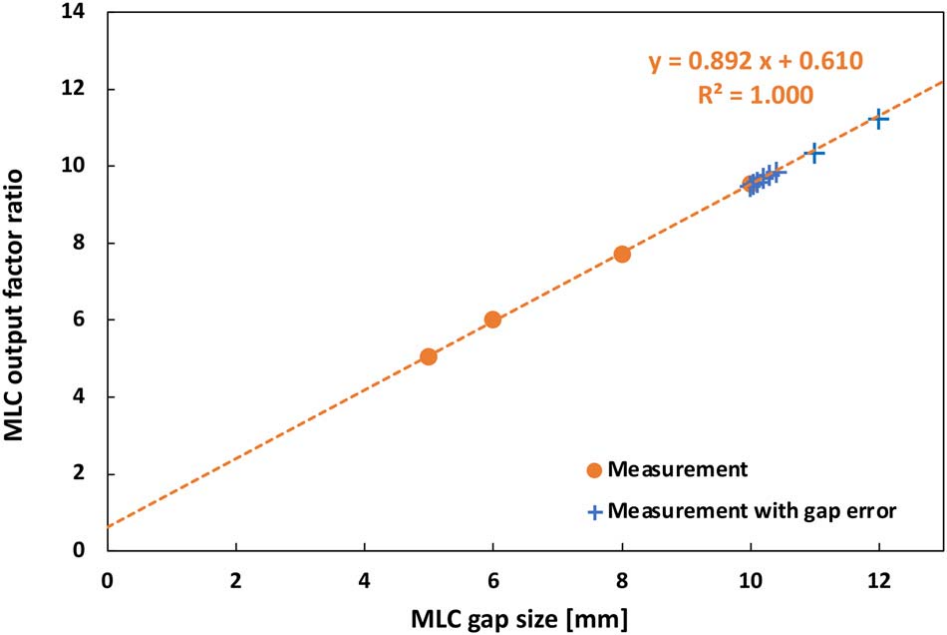
Correlation between the MLC gap width and the measured MLC output ratio.

The coefficient of variation for the measurement was 0.2%. The reproducibility of the results was also confirmed. Figure 11 shows the correlation between the MLC gap width and measured MLC output ratio. Circle plots indicate the MLC output ratios for widths of 5, 6, 8 and 10-mm. A linear fitting was performed on the data. Cross plots indicate the 10 mm width MLC output ratio with the intentional gap error. The results were compared with the MLC output calculated using the linear correlation, and the average difference was −0.39% (Table 4). This correlation is considered to be effective for detecting the MLC gap-width error.

#### IMRT commissioning

**Fig. 12:**
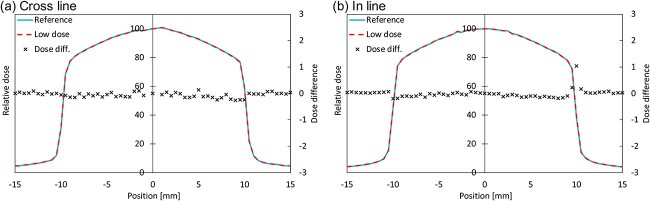
Cross-line (a) and in-line (b) profiles measured by IC profiler.

Figures 12 (a) and (b) show the cross- and in-line profiles for low-dose delivery and reference, respectively. All profiles were normalized to the central axis. The profiles for low-dose delivery were in good agreement with the reference. The deviation of the symmetry and dose at CAX are listed in Table 5. The averages of the GPR and point DD was 98.6 ± 0.9% and − 0.3 ± 1.1%, respectively.

#### End-to-end testing

**Fig. 13:**
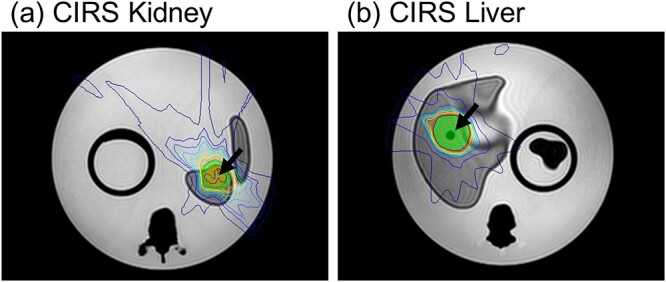
Initial plan for kidney and liver cancer on CIRS phantom.

The initial plans for kidney cancer and liver cancers are shown in Fig. 13. Black arrows indicate the tumor volumes. During adaptation, the ATP and ATS plans were optimized and calculated for each cancer. Table 6 shows the DDs between the measurements and calculations. The DDs for MU2net and TPS were less than 3% and 2%, respectively. Gamma analysis was performed. All GPR results were > 95%.

**Table 2:** Difference between the TPS and MU2net calculations for single beams

MLC margin	TPS calc dose	MU2net calc dose	Diff.
3 mm	0.800	0.838	4.75%
5 mm	0.815	0.844	3.56%
10 mm	0.849	0.864	1.77%

#### Result of patient-specific QA

The GPRs for all patients treated at our institute was evaluated. All cases exceeded 95%, and the average GPR was 97.9%. All measured IMRT plans were within the recommended tolerances of the AAPM Task Group 218. The point DD between the measurement and calculation was −0.6 ± 1.1%.

**Table 3:** Difference between the TPS and MU2net calculations for four-field conventional and IMRT plans

Plan	Gantry angle	TPS calc dose	MU2net calc dose	Diff. %
Four-field conventional plan	0	0.830	0.854	2.89
	90	0.736	0.761	3.40
	180	0.800	0.838	4.75
	270	0.729	0.758	3.98
	***Composite***	***3.095***	***3.203***	***3.49***
Seven-field IMRT plan	154	1.469	1.490	1.43
	103	1.514	1.551	2.44
	51	1.675	1.753	4.66
	0	1.473	1.506	2.24
	309	1.271	1.354	6.53
	257	1.506	1.572	4.38
	206	1.508	1.608	6.63
	***Composite***	***10.416***	***10.832***	***3.99***

## DISCUSSION

Unity provides the images with the superior contrast for tumor visualization, and the ability to create online adaptive plans that consider daily anatomical changes. The MRgOART is likely to improve the safety and efficacy of treatment; however, treatment must be commissioned accurately before clinical use. A few articles have been published on the commissioning of Unity, and Kerkmeijer *et al*. reported that the experience-sharing is desirable [17]. This study provides commissioning results including novel procedures.

**Table 4:** Difference between the measured value and the expected value of the gap errors

Gap error [mm]	Measured value	Expected value	Difference [%]
0.1	9.568	9.599	−0.32
0.2	9.671	9.688	−0.17
0.3	9.755	9.777	−0.22
0.4	9.833	9.866	−0.33
1	10.330	10.400	−0.67
2	11.217	11.290	−0.64

QA of the medical accelerator was recommended by the AAPM Task Group 142 [18]. The size of the radiation isocenter of the Unity system was 0.41 mm, which is smaller than standard linacs [19]. It was confirmed that the size was sufficient to offer SBRT because it was within the tolerance recommended by the AAPM Task Group 142. The positional accuracy of the single-isocenter stereotactic radiosurgery and stereotactic body radiation therapy technique to treat multiple lesions was verified, and the authors reported an acceptable distance from the center of the mechanical field to the machine isocenter [20, 21]. In the Unity system, the patient cannot be shifted to the isocenter; rather, the position of the MLCs is changed through the ATP and ATS procedures [15]. Hence, the accuracy of dose delivery to the off-center target is very important. Our off-center radiation isocenter results were within 1.0 mm; however, we observed that a trend for the

larger result to increase with the distance. Therefore, it is recommended that our QA procedure be performed during commissioning. To the best of our knowledge, this is the first off-center radiation isocenter verification performed using the Elekta Unity system. In addition, leaf pairs further off-center will likely be more frequently used than those of standard linac plans and, it is also important to verify the positional accuracy of all MLCs. We added a film-based fence test to verify the positional accuracy of all the leaves. The film- and MVI-based fence tests yielded similar results and were found to be within acceptable limits [22]. Our film-based method could not evaluate 80 pairs of MLCs (evaluate 70 pairs of MLCs), but the method performed in previous work requires special jigs and is not versatile [6].

The non-standard geometry and the effect of magnetic fields complicate the reference dosimetry calibration. For dosimetry calibration, TPR_20,10_ was used as the beam quality indicator. The measured TPR_20,10_ was 0.705, which was approximately the same as the values of 0.704 and 0.705 reported by Snyder *et al*. and Powers *et al.*, respectively [6, 7]. In previous works, the calibration was performed at a gantry angle of 90° instead of 0°. Independent validation showed that an agreement within ±5% was considered a satisfactory result.

Monaco TPS handled effects such as the Lorentz force and electron return effect, and the TPS calculation showed good agreement with the measured profiles. The average of point DD was 0.11%. In the future, it may be required to compare the measured beam data between facilities own Elekta Unity to provide representative beam data. The Unity system has a cryostat and a couch; therefore, dosimetry is complicated by non-uniform materials. Because the attenuation correction function varies significantly depending on manufacturing errors, such as welding, it is necessary that the measurement data be entered into the TPS. Measurement of gantry angle dependency, including the dosimetric impact that depends on the cryostat and couch attenuation was performed, and the difference between the calculation and measurement was within −0.2%. We find that this is a good result compared to the result of Snyder *et al*. [6]. Beam profiles irradiated with 3 MU were measured using the IC-profiler to evaluate the reproducibility. These findings were confirmed through visual and quantitative evaluations. IMRT plans were measured using ArcCHECK, and all results were within the tolerance of the AAPM Task Group 218 [14]. The average point DD between the measurement and calculation was −0.3%. From the above results, we confirmed that the calculations and measurements were in agreement for 3D conventional radiotherapy and IMRT plans. The results of the patient specific QA in clinical use do not show any significant change from the IMRT commissioning results.

For the irradiated fields with maximum areas of 15.0 × 15.0 cm^2^ at the isocenter, the agreement between the MU2net calculation and measurement was acceptable. However, MU2net is not suitable for calculating the dose for larger field sizes. Our results tended to be similar to those in the reference [23]. The calculation errors may have occurred for the following reasons: (1) the difference between our attenuation coefficient and modeled one; (2) the Clarkson algorithm-based calculation; and (3) non-magnetic field environment. The validation results for the inhomogeneous phantom were within the action level recommended by AAPM Task Group 219 [9]. This validation should serve as the baseline for any additional analysis, and should be verified again after any software updates.

QA tests for the dosimetry of the MLC delivery extend the QA tests for MLC positional accuracy. In particular, the MLC output ratio provides a highly sensitive measure of the changes in the position of the MLCs during delivery. Therefore, the baseline changes may confirm a reproducible dosimetry for the S-IMRT delivery. Our results show that an MLC gap width with an error of 0.3 mm results in a dose error of approximately 2.9%. This result is the same as that of reference [8].

End-to-end tests of the CIRS phantom for the ATP and ATS workflows yielded measurements that were within 2% of the calculation. The results of gamma analysis of these plans were greater than 98%. In MRgOART, it is necessary to use a phantom made of a material that is visible in both MR and CT images. Therefore, the use of this phantom is desirable. It is also an important commissioning process for determining the workflow of the MRgOART.

**Table 5:** Result of the symmetry and dose reproducibility

	Symmetry		Dose at CAX
	X axis	Y axis	
Reference	101.8	101.1	-
Avg. (Low dose)	101.9	101.1	3.00
SD (Low dose)	0.08	0.07	0.004

**Table 6:** End-to-end test results

	Liver cancer		Kidney cancer	
	ATP	ATS	ATP	ATS
Dose difference for MU2net [%]	−0.4	−0.2	−2.2	1.1
Dose difference for TPS [%]	−0.7	−0.5	2.0	1.8
GPR [%]	99.1	98.4	99.6	100

## CONCLUSION

We reported the results of mechanical and dosimetric performances of Elekta Unity, and a novel off-WL test procedure was developed for verifying the accuracy of non-isoncentric beam irradiation. The results of the off-WL test were within 1 mm, the criteria required for stereotactic radiotherapy. Moreover, an independent dose calculation for the inhomogeneous phantom was performed, and we confirmed that the results were within the criteria of AAPM Task Group 219. Our results should be beneficial for facilities that install such equipment.

## CONFLICT OF INTEREST

Masato Tsuneda, Kota Abe and Yukio Fujita have endowed chairs funded by Elekta K. K.

## Funding

This work was supported by JSPS KAKENHI (grant number JP18K15607).
